# Interleukin-6 neutralization alleviates acute exacerbation-like disease in a model of cigarette smoke-induced pulmonary inflammation

**DOI:** 10.1186/1476-9255-10-S1-P33

**Published:** 2013-08-14

**Authors:** John Kubera, Katherine Hammerman, Cara MM Williams, Cedric Hubeau

**Affiliations:** 1 Inflammation and Remodeling, Pfizer Cambridge, Massachusetts, 02140, USA; 2 Drug Safety R&D, Pfizer Cambridge, Massachusetts, 02140, USA

## 

Increased systemic and pulmonary levels of interleukin (IL)-6 have been associated with the severity of acute exacerbations and accelerated decline of lung function in COPD patients. The demonstration that IL-6 plays a pivotal role in AE-related pulmonary symptoms and therefore represents a therapeutic target for the treatment of COPD, remains elusive. We used a murine model where C57BL/6 female mice are exposed to cigarette smoke (CS) twice daily through a nose-only system, while being punctually challenged intranasally with poly I:C, a synthetic ligand for Toll Like Receptor-3 (TLR3). This protocol recapitulates several aspects of pulmonary inflammation as seen in acute exacerbations of COPD, including prominent airway neutrophilia as well as increased levels of type I interferon, GM-CSF, IL-6, KC, MIP-1 alpha, RANTES, and TNF-alpha in bronchoalveolar lavage (BAL) samples. Using this model, IL-6 deficient mice showed a susceptibility to CS-induced pulmonary inflammation that, overall, was comparable to that found with wild-type (WT) control mice. In contrast, using the same model with WT mice treated intraperitoneally with IL-6 neutralizing antibodies (rat IgG1, clone MP5-20F3, 25 mg/kg thrice weekly) diminished blood counts of lymphocytes (p=0.0070) and monocytes (p=0.0091), while this treatment also depleted BAL levels of IL-6 (p=0.0002) and reduced BAL levels of KC (p=0.0220). Total BAL cellularity was found to be largely decreased (p<0.0001) as well as BAL numbers of neutrophils (p=0.0031), lymphocytes (p<0.0001) and macrophages (p<0.0001), while inflammatory infiltrates seemed reduced in lung tissue sections from treated mice. Our results show that the neutralization of IL-6 largely abrogates pulmonary inflammation in CS-exposed mice, and therefore indicate that IL-6 may be a valid therapeutic target for the treatment of COPD, in particular in episodes of acute exacerbation.

